# Systemic Inflammation in Chronic Obstructive Pulmonary Disease: May Adipose Tissue Play a Role? Review of the Literature and Future Perspectives

**DOI:** 10.1155/2010/585989

**Published:** 2010-04-20

**Authors:** Ruzena Tkacova

**Affiliations:** Department of Respiratory Medicine and Tuberculosis, Faculty of Medicine, P. J. Safarik University, L. Pasteur Teaching Hospital, Kosice 041 90, Slovakia

## Abstract

Chronic obstructive pulmonary disease (COPD) is a major cause of morbidity and mortality worldwide. Low-grade systemic inflammation is considered a hallmark of COPD that potentially links COPD to increased rate of systemic manifestations of the disease. Obesity with/without the metabolic syndrome and cachexia represent two poles of metabolic abnormalities that may relate to systemic inflammation. On one hand systemic inflammatory syndrome likely reflects inflammation in the lungs, i.e. results from lung-to plasma spillover of inflammatory mediators. On the other hand, obesity-related hypoxia results in local inflammatory response within adipose tissue *per se*, and may contribute to elevations in circulatory mediators by spillover from the adipose tissue to the systemic compartment. The extent to which systemic hypoxia contributes to the adipose tissue inflammation remains unknown. We assume that in patients with COPD and concurrent obesity at least three factors play a role in the systemic inflammatory syndrome: the severity of pulmonary impairment, the degree of obesity-related adipose tissue hypoxia, and the severity of systemic hypoxia due to reduced pulmonary functions. The present review summarizes the epidemiological and clinical evidence linking COPD to obesity, the role of adipose tissue as an endocrine organ, and the role of hypoxia in adipose tissue inflammation.

## 1. Introduction

According to the WHO, chronic obstructive pulmonary disease (COPD) is one of the most prevalent diseases, expected to move to the 3rd leading cause of mortality in 2020 [1]. COPD is characterized by poorly reversible airflow limitation that is usually progressive and associated with an abnormal inflammatory response of the lungs to noxious particles or gases, particularly cigarette smoke [2]. Nevertheless, the pathological mechanisms and clinical manifestations of COPD are not restricted only to pulmonary inflammation and airway remodeling [3]. In contrast, over the last decade, the recognition of COPD as a systemic disease has developed. The best recognized systemic manifestations of COPD include systemic inflammation, cardiovascular comorbidities, cachexia and muscle dysfunction, osteoporosis, anemia, and clinical depression and anxiety [4, 5]. Chronic comorbidities affect health outcomes in patients with COPD, including mortality. In fact, the majority of patients with COPD die of nonrespiratory disorders such as cardiovascular diseases or cancer [6].

Low-grade systemic inflammation can be defined as a two- to fourfold elevation in circulating levels of proinflammatory and anti-inflammatory cytokines, naturally occurring cytokine antagonists, acute phase proteins, as well as minor increases in counts of neutrophil and natural killer cells [7]. Systemic inflammation is considered a hallmark of COPD and one of the key mechanisms that may be responsible for the increased rate of comorbidities, including cardiovascular complications in COPD [8]. Obesity with/without the metabolic syndrome on one hand and cachexia on the other represent two poles of metabolic abnormalities that may relate to systemic inflammation. Respirologists are routinely challenged by the presentation of obesity concurrently with COPD, particularly in GOLD stages 1 and 2 [9]. Obesity and cachexia are clinically significant and challenging in both clinical practice and translational research of systemic manifestations of COPD [9, 10]. 

Increased expression and secretion of proinflammatory adipokines resulting from obesity and/or hypoxia in patients with COPD may represent a contributing mechanism aggravating the overall systemic inflammatory pattern in this multicomponent disease. Although the extent to which adipose tissue production and release of inflammatory cytokines contributes to the chronic systemic inflammatory syndrome in COPD is not yet well defined, several stimulating ideas can be derived from experimental studies aimed to unravel the effects of hypoxia in adipocyte cell cultures, from animal hypoxic models, and from disorders other than COPD. The purpose of this review is to summarize recent advances and to outline future perspectives in the search for the evidence of the potential role of adipose tissue in the systemic inflammation in patients with COPD.

## 2. Search Strategy

A search of scientific literature was performed to identify relevant studies on the topic. Following electronic databases were searched: Cochrane Database of Systematic Reviews, Medline via PubMed, and Google Scholar. Specific keywords, terms, and their combinations including searches with MESH terminology (i.e., *“obesity and COPD and review*”) were used in three areas: (1) terms related to clinical manifestations of COPD: *“chronic obstructive pulmonary disease”, “adipose tissue”, “morbidity”, “mortality”, “clinical outcomes”, “exacerbation”, “comorbidities”, “systemic manifestations”, “obesity”, “cachexia”, “weight loss”; *(2) terms related to systemic inflammation: *“systemic inflammation”, “hypoxia”, “inflammatory cytokines”, “adipokines”, “transcription factors”, “gene expression”, “tumor necrosis factor”, “interleukin”, “hypoxia-inducible factor”, “nuclear factor-kappa B*”; (3) terms related to metabolic state: *“insulin resistance”, “nutrition”, “body composition”, “fat mass”, “fat-free mass”, “resting energy expenditure”*. The searches were limited to papers published in English-language journals in the last 15 years. In addition, we searched relevant guidelines and latest editions of internal and respiratory medicine textbooks. Given the journal space limits, we restrict the present review to the most robust, relevant, and important studies in the reviewed topic.

## 3. Systemic Inflammation in COPD

### 3.1. COPD—Chronic Systemic Inflammatory Syndrome

Recently, Fabbri and Rabe [11] proposed an overreaching approach to diagnosis, assessment of severity, and management of COPD and its frequent comorbidities that aims not to restrict the diagnostic approach to COPD alone but to search for the signs of the more general disorder termed “chronic systemic inflammatory syndrome”. The rationale for this proposal described in details in Lancet [11] can be shortly summarized as (a) systemic inflammation as the most likely key common mechanism by which major risk factors such as smoking, hyperlipidemia, obesity, and hypertension lead to chronic diseases [12] including COPD, (b) the presence of at least three chronic medical conditions in almost half of all people aged 65 years or older, with comorbid conditions accounting for more than 50% of health-care resources in patients with COPD [13]. 

The epidemiological, pathogenetic, and clinical evidence linking COPD to chronic systemic inflammatory syndrome is rapidly growing. Even during stable COPD, increases in a number of inflammatory proteins were described in the systemic circulation including C-reactive protein (CRP) [7, 14–16], tumor necrosis factor-alpha (TNF-*α*), interleukin (IL)-6 [17] and IL-8 [18]. Small but significant increases in circulating levels of both the soluble TNF receptors 55 and 75 (sTNF-R55 and sTNF-R75) [18], IL-10 [19] and IL-18 [20] have also been reported in such patients. Importantly, epidemiological studies suggest relationships between circulatory inflammatory mediators and reductions in pulmonary functions reflected by decreases in forced expiratory volume in one second (FEV_1_). Such relationships were observed between pulmonary functions and circulatory CRP [7, 21] or IL-6 [22]. Moreover, some evidence suggests that the systemic proinflammatory state is likely not counterbalanced by up-regulation of anti-inflammatory proteins. Indeed, Dentener et al. [14] documented that levels of the anti-inflammatory mediator-soluble interleukin 1 receptor II (sIL-1RII) were similar in patients with stable COPD compared to healthy subjects, despite markedly increased sTNF-R55. It is important to underline that significant repeatability of circulatory inflammatory biomarkers such as IL-6, TNF-*α*, and CRP over twelve-month period, with a robust and repeatable association between IL-6 and CRP levels was recently demonstrated [23].

COPD exacerbations are associated with further increases in markers of both bronchial and systemic inflammation over and above levels present during the stable phase of the disease [24–26]. In addition, the rate of decline of inflammatory mediators such as CRP during exacerbations of COPD is significantly diminished compared to other inflammatory conditions such as pneumonia, with only 6% of COPD patients responding by CRP reductions on the second day of hospitalization following antibiotic administration [27]. 

### 3.2. Origin of Systemic Inflammation in COPD

Might COPD-associated chronic inflammatory syndrome reflected by increases in circulatory concentrations of inflammatory proteins relate to concurrent obesity or cachexia? In COPD, two main sources of inflammatory mediators may be considered: lungs and peripheral organs including adipose tissue. Indeed, there is a long-lasting debate whether, in patients with COPD, the local inflammation in the pulmonary compartment spills over into the circulation or rather there is an enhanced production of inflammatory mediators in non-pulmonary compartment as well. Approaching this question is rather challenging since plasma biomarkers such as CRP, TNF-*α*, interleukins, and fibrinogen are clinically relevant [28, 29] but are synthesized predominantly in the liver and, thus, their relationship with COPD and lung functions remains uncertain [30, 31]. Analysis of the origin of systemic inflammation in COPD might require different approach: to study either lung-specific proteins (i.e., pneumo-proteins) whoes plasma concentrations are largely determined by the rate of synthesis and translocation across the lung-blood barrier, and/or adipose-tissue specific proteins in association with measurements of arteriovenous differences in the concentrations of the respective inflammatory mediators (Sin DD, personal communication). 

#### 3.2.1. Lung-to-Blood Translocation of Inflammatory Mediators

Recently, the concept of systemic inflammation as a consequence of spillover of inflammatory mediators from the lungs to the systemic compartment in COPD has been broadly discussed [32–34]. Several lines of evidence suggests that smoking *per se* and/or COPD increase permeability of pulmonary vessels, and therefore contribute to the spill-over of inflammatory mediators from the pulmonary to the systemic compartment [35–38]. First, in airway cell line in vitro, exposure to cigarette smoke transiently decreases transepithelial resistance in association with increases in macromolecular permeability [37]. Second, experimental studies in mice suggest that lung injury induced by bleomycin exposure leads to increased leak of surfactant protein D (SP-D), a lung specific protein from the pulmonary to the systemic compartment [39]. Also, lung-to-blood translocation of IL-6, a primary inflammatory cytokine, has been documented following exposure to endotoxin [40]. Third line of evidence on the lung-to-blood translocation of inflammatory mediators comes from human studies. Kennedy et al. [35] identified higher alveolar-capillary membrane permeability in smokers compared to nonsmokers using the most direct measurement of the blood-gas barrier permeability by radioactive-labeled hydrophilic solute ^99m^Tc-labelled diethylenetriaminepentaacetic acid (DTPA) following its inhalation and clearance from the alveoli into the pulmonary capillary blood. Moreover, Wollmer and Evander [36] demonstrated that lung clearance of DTPA is inversely related to FEV_1_ such that subjects with the lowest pulmonary function had the highest values of permeability. Conversely, improvements in FEV_1_ following therapy with inhaled steroids were accompanied by significant reductions in alveolar-capillary membrane permeability in symptomatic patients with COPD [41]. Also, reductions in serum levels of several inflammatory mediators including lung-specific proteins such as surfactant protein-D were observed in patients with moderate-tosevere COPD following therapy with inhaled corticosteroids [42, 43].

#### 3.2.2. Increases in Inflammatory Cytokine Production and Release from Peripheral Tissues

In conditions other than COPD, large number of studies suggest that both liver and adipose tissue express a wide variety of proinflammatory mediators that are released to the systemic circulation, and have potential systemic effects. In the pathogenesis of low-grade subclinical inflammation in obesity, the importance of adipokines released from adipose tissue is increasingly recognized. Indeed, visceral fat is an important source of IL -6, and portal vein IL-6 levels relate to arterial C-reactive protein levels [44]. Nevertheless, despite observed links between visceral adiposity and systemic inflammation, no studies addressed the question regarding adipose tissue contribution to systemic inflammation in patients with COPD.

#### 3.2.3. A Novel Concept of Systemic Inflammation Origin in COPD: Dependence on Pulmonary Impairment and Obesity

**Figure 1:**
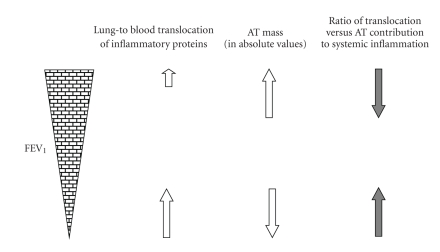
Concept of differential contribution of lung-to-blood translocation of inflammatory proteins versus adipose tissue inflammation to the overall systemic inflammatory phenotype in COPD. We suggest that in patients with mild COPD and concurrent obesity, adipose tissue (AT) is the key contributor to systemic inflammation, whereas in those with severe COPD, lung-to-blood translocation of inflammatory proteins plays the major role.

Three lines of evidence, that is, experimental studies, studies in humans involving measurements of lung vasculature permeability, and treatment studies in COPD with inhaled corticosteroids support the concept that pulmonary inflammation is the prevailing mechanism contributing to systemic inflammation in such patients. Nevertheless, when interpreting the above studies, one crucial factor needs to be taken into account: the severity of lung structure/function impairment. Since highest permeability of pulmonary vessels occurs in subjects with the lowest FEV_1_ [36], and inhaled corticosteroids reduced systemic inflammation in patients with at least moderate degree of bronchial obstruction [42, 43], the mechanism of inflammatory proteins spill-over from the pulmonary to the systemic compartment is likely to have the highest relevance in patients with more pronounced pulmonary impairment. Nevertheless, lung-to-blood translocation of inflammatory mediators may not be prominent in subjects with mild form of the disease. In contrast, the prevalence and degree of obesity are significantly higher in patients with mild compared to more advanced COPD (see further) [9, 45]. Therefore, it is reasonable to assume that obesity-related adipose tissue inflammation likely represents a significant contributor to the overall systemic inflammatory profile in patients with mild form of the disease with concurrent obesity (Figure 1). The hypothesis that adipose tissue may contribute to the overall systemic inflammatory phenotype in patients with early stages COPD with obesity or relative abundant fat mass is novel [10]. Therefore, we discuss further essential issues related to body composition in COPD, endocrine function of adipose tissue, and the role of hypoxia in adipose tissue inflammation.

## 4. Body Composition in COPD

In patients with COPD, obesity is characterized by an absolute abundance of fat mass (FM), similar to other diseases associated with excessive adiposity (i.e., metabolic syndrome, hypercorticism, etc.). The prevalence of abdominal obesity measured by waist circumference is almost twice as high as in age- and sex-matched controls [46]. Patients with normal body mass index (BMI) display either physiological or reduced ratio of fat-free mass (FFM) to FM. Importantly, selective wasting of FFM (i.e., loss of muscle mass) occurs in about 10% of COPD patients with normal BMI, resulting in a relative or absolute increase in FM [47–49]. Nevertheless, cachectic patients with COPD experiencing reductions in their body weight below the physiological range suffer from both loss of FFM in association with the loss of FM [50].

### 4.1. Epidemiology of Obesity in COPD

Obesity has emerged as an important risk factor for respiratory disorders, and a link between obesity and/or metabolic syndrome and COPD is increasingly recognized [10, 51, 52]. The prevalence of obesity is the highest among patients with milder forms of the disease (GOLD Stages 1 and 2), and the lowest in patients with the most severe lung function impairment in Stage 4 [8, 45]. Marquis et al. [46] demonstrated the presence of one or more components of the metabolic syndrome in almost 50% of COPD patients. Furthermore, in a recent population-based survey that involved 7,358 adults aged ≥50 years, the risk of metabolic syndrome was higher in those with airflow obstruction compared to those without [53]. In the Tucson prospective cohort study, prevalence of BMI ≥28 kg/m^2^ was significantly higher in patients with chronic bronchitis compared to controls (25 versus 16%) [54]. Furthermore, two large epidemiological studies identified increased prevalence of obesity among patients with COPD than in the general population. In Northern California (USA), 54% of patients with COPD were obese and had BMI ≥30 kg/m^2^ compared to 20–24% prevalence of obesity in the general population [55]. In the European cohort of COPD patients in Netherlands, obesity prevalence of 18% was reported [9] in contrast to only 10–12% prevalence in the general cohort of residents in the same region [55]. So far the largest and the most convincing study examined 121,965 men and women in France, and demonstrated an independent association between lung function impairment and the metabolic syndrome after correction for age, sex, smoking status, BMI, physical activity, and cardiovascular disease history [52]. Interestingly, abdominal obesity was the strongest predictor of lung function impairment in both men and women [52]. Similar associations between central obesity and airflow obstruction that were independent of smoking status were observed recently also in a large Chinese cohort [53]. 

### 4.2. Putative Mechanisms

**Figure 2:**
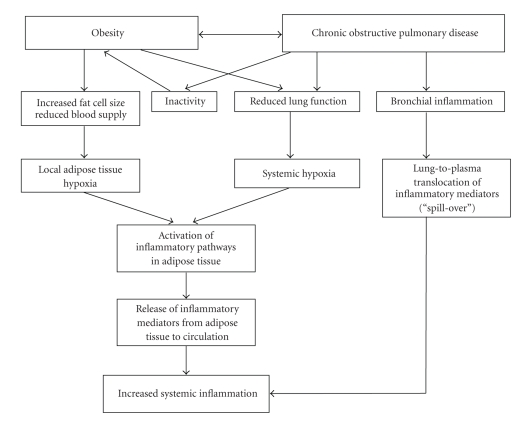
Proposed mechanistic links between the combined effects of obesity-related local adipose tissue hypoxia, reduced lung function-related systemic hypoxia, and bronchial inflammation-related increased lung-to-plasma translocation of inflammatory mediators on systemic inflammatory profile in patients with COPD.

The nature of the association between obesity and COPD has not been fully elucidated [51]. Nevertheless, several pathogenetic mechanisms may link these two conditions (Figure 2). First, as indicated before, systemic inflammatory syndrome is a hallmark of both conditions obesity and COPD. Second, reduced physical inactivity is frequently observed in patients with COPD [56]. On one hand, airflow limitation limits exercise performance and reduces physical activity, and consequently increases propensity to weight gain in patients with COPD. On the other hand, obesity further compromises pulmonary functions [10], and therefore represents additional factor contributing to the patient's inactivity (Figure 2). Importantly, a recent study by Watz et al. [57] demonstrated that the coexisting obesity and metabolic syndrome are associated with both increases in systemic inflammatory markers and reduced physical activity independently of lung function impairment. In reverse, regular physical activity modifies smoking-related lung function decline and development of COPD [58].

### 4.3. Clinical Implications of Obesity in COPD

High adiposity and fat tissue accumulation impair pulmonary functions and exercise performance [50, 51, 55]; this topic has been recently extensively reviewed [10]. Additionally, obesity and the presence of metabolic syndrome are related to increased insulin resistance in overweight and obese COPD patients [59, 60]. The study by Bolton et al. [59] suggests that insulin resistance is aggravated by both, high BMI and increases in circulatory inflammatory mediators such as IL-6 in these patients. Indeed, inflammatory mediators TNF-*α*, IL-6, and leptin were significantly higher while plasma adiponectin levels were reduced in the presence of excess weight in COPD patients [51]. 

In patients with COPD, the relationship between body weight and mortality risk is not unidirectional. In earlier stages of this disease, low-grade systemic inflammation related to visceral obesity likely represents one of the key factors contributing to increased risk of comorbidities such as cardiovascular complications and type 2 diabetes. Therefore, similar to the metabolic syndrome and type 2 diabetes, obesity in COPD patients with GOLD Stages 1 and 2 severity may contribute to their increased cardiovascular and all-cause mortality [10]. In contrast, the relative mortality risk is reduced in overweight patients with severely deteriorated lung functions in GOLD Stages 3 and 4 [61, 62], a condition described as “obesity paradox”. Of note, obesity paradox has been repeatedly reported not only in severe COPD but also in other serious conditions associated with wasting and high mortality risk such as malignancies [63], chronic heart failure [64], renal failure [65], and AIDS [66].

## 5. Inflammatory Mediators and Adipose Tissue

### 5.1. Adipose Tissue as an Endocrine Organ

Adipose tissue is currently viewed as a highly dynamic endocrine organ that is involved in a wide range of metabolic processes [67–70]. Within adipose tissue, adipocytes, macrophages, and endothelial cells synthesize and release a large number of a diverse group of proteins involved in several functional categories such as inflammation and acute-phase response, immunity, insulin sensitivity, angiogenesis, blood pressure control, lipid metabolism and hemostasis [69], recommend that the term “adipokine” be universally used to describe proteins synthesized by and secreted from adipocytes. Adipocytes express and secrete a variety of adipokines including cytokines, growth factors, adiponectin, resistin, adipsin, leptin, acylation stimulating protein, plasminogen activator inhibitor-1 (PAI-1), lipoprotein lipase, and components of the renin-angiotensin system which may exert local (autocrine/paracrine) and systemic (endocrine) effects [67–69].

### 5.2. TNF-*α* and  Interleukins

Marked increases in the expression of TNF-*α* were identified in obese mouse models [71], and were linked to obesity and insulin resistance. Nevertheless, although TNF-*α* secretion is increased in enlarged adipocytes, TNF-*α* *per se* has proapoptotic properties [72]. A number of adipocyte-abundant genes (GLUT4, hormone sensitive lipase, long-chain fatty acyl-CoA synthase, adipocyte complement-related protein of 30 kDa, and transcription factors CCAT/enhancer binding protein-alpha, receptor retinoid X receptor-alpha, and PPAR*γ*) are significantly down-regulated by TNF-*α* [73]. Correspondingly, exposure of adipocytes to TNF-*α* results in reduced GLUT4 and several insulin signaling proteins levels (insulin receptor, insulin receptor substrate 1, and protein kinase (B). NF-*κ*B is activated within 15 minutes of TNF-*α* addition. The absence of NF-*κ*B activation leads to a release of more than 98% of the genes normally suppressed by TNF-*α*, and, on the other hand, to a suppression of 60–70% of the genes whose expression is physiologically induced by TNF-*α* [73]. Taken together, changes in adipocyte gene expression induced by TNF-*α* could lead to insulin resistance, and NF-*κ*B seems to be an obligatory mediator of most of these TNF-*α* responses. 

As much as a third of total circulating concentrations of IL-6 has been estimated to originate from adipose tissue [74], and circulatory concentrations of IL-6 were shown to be related to BMI. TNF-*α* is a potent stimulus for IL-6 synthesis in adipocytes: a 60-fold increase in IL-6 production in differentiated 3T3-L1 adipocytes was observed after TNF-*α* exposure [75], in association with enhanced shedding of sTNF-RII receptors from the adipose tissue [74]. IL-6 decreases adipose tissue LPL activity, and has been implicated in lipolysis and fat depletion taking place during cancer cachexia [76]. Like TNF-*α*, IL-6 has potent effects on adipose tissue, as evidenced by the fact that neutralization of this cytokine decreases progressive loss of adipose tissue during cachexia [77]. 

IL-8 is also highly expressed and produced in human adipocyte tissue, and similarly to IL-6, TNF-*α* represents a potent stimulus for the production and release of IL-8 from adipocytes. In obese subjects, positive correlations between circulating TNF-*α* and IL-8 were demonstrated [78]. Nevertheless, no data are available either on the potential link between adipose tissue expression of IL-6 and IL-8, and their respective circulatory levels in patients with COPD.

### 5.3. Leptin and Adiponectin

Leptin, a protein synthesized by white adipose tissue and encoded by the *ob* gene, plays an important role in energy balance. Growing body of evidence suggests that leptin may have an important role in up-regulating the inflammatory system: not only does TNF-*α* promote release of leptin from adipose tissue [79] but leptin may *per se* up-regulate expression of proinflammatory cytokines [80]. Plasma leptin correlates with BMI, and weight loss reduces its circulatory concentrations in a variety of human disorders associated with obesity [81]. Nevertheless, only a few studies analyzed associations between lung function impairment and circulatory leptin. In 2808 nonobese individuals, Sin and Man [82] demonstrated that those with impaired lung function have raised serum leptin levels. In another study, Creutzberg et al. [83] documented impairments of normal leptin feedback mechanisms during exacerbations of COPD suggesting that elevated leptin concentrations might represent an up-regulation of leptin mRNA in adipose tissue resulting in an enhanced leptin production in such patients. So far the only adipocyte-derived hormone that is known to exert anti-inflammatory effects is adiponectin, and its expression is reduced in obesity [84, 85]. Acute exacerbations of COPD are associated with increases in serum leptin levels and the leptin/adiponectin ratio, and these elevations were related to serum IL-6 and TNF-*α* [84]. In turn, adiponectin levels increased at resolution of COPD exacerbation [86].

### 5.4. Adipocyte Tissue Hypoxia in Obesity: Effects on Inflammatory Mediators

The expression and/or secretion of inflammatory molecules are increased in the adipose tissue of obese insulin-resistant individuals. In contrast to small adipocytes, enlarged adipocytes in conditions associated with obesity express proinflammatory and anti-inflammatory factors with a shift towards dominance of proinflammatory adipokines [87]. Elevated levels of IL-6 were detected in obese women, and were reduced after weight loss [88]. In addition, increased expression of inflammatory mediators such as TNF-*α* [71, 89], PAI-1 [90], and leptin [89] was detected in human obesity. 

Chronic low-grade adipose tissue inflammation in obesity may represent a specific response to relative hypoxia of adipocytes [69]. Several factors may contribute to cell hypoxia within adipose tissue in association with high adiposity: (a) blood flow per unit adipose tissue mass is reduced in obese humans [91] resulting in decreased blood supply to the tissue; (b) large adipocytes are further from the vasculature than the normal diffusion distance for O_2_ (150 versus 100 *μ*m). Therefore, clusters of adipocytes become distant from the vasculature as cell size increases resulting in their relative hypoxia and increased inflammatory response which serves to increase blood flow and to stimulate angiogenesis [69]. 

Adipocyte tissue hypoxia has detrimental effects on cell metabolism and function, as evidenced by studies in vitro and animal models. Studies in vitro have shown that hypoxia results in enhanced TNF-*α* production, increased expression of PAI-1, and reduced adiponectin and peroxisome proliferator-activated receptor gamma (PPAR*γ*) expression [92, 93]. In addition, animal models in obese mice demonstrated occurrence of hypoxia in adipose tissue in association with increased expression of proinflammatory adipokines [93]. Hypoxia-induced factor (HIF)-1*α*  represents the key hypoxia-sensing protein in most tissues. Not only does it play a central role by signaling the presence of hypoxia to the transcriptional systems in the nuclei of all cells but it also activates a number of target genes whose products are involved in inflammatory responses such as IL-6, TNF-*α*, macrophage migration inhibitory factor, vascular endothelial growth factor, tissue inhibitor of metalloproteinases-1, and monocyte chemotactic protein [94, 95]. Transcription factors such as NF-*κ*B and sterol regulatory element-binding protein (SREBP) are downstream molecules of HIF-1*α* signaling pathways that in turn regulate production of TNF-*α* and IL-6 [96]. The number of identified genes with demonstrated responsiveness to HIF-1*α* is steadily growing, and to date more than 70 genes have been described as targets of this molecule [94]. These genes encode proteins involved in energy metabolism, cell proliferation, apoptosis, and angiogenesis [97–99]. Genes whose expression is up- or down-regulated in adipocytes by hypoxia have been recently extensively reviewed elsewhere [100].

### 5.5. Systemic Hypoxia in Respiratory Disorders

Significant inverse relationships were observed between PaO_2_ and circulating TNF-*α* and staff-R levels in patients with COPD [101]. Lipid peroxidation products are increased, and antioxidative enzymes are reduced in the circulation of patients with COPD at rest and during exercise [102], and increase even further during exacerbations of the disease [103]. In addition, an association between markers of systemic inflammation and markers of oxidative stress during exacerbations of COPD was described [103]. However, no studies were undertaken until now to analyze adipose tissue inflammation in patients either with stable COPD or during the disease exacerbations. It is reasonable to hypothesize that increases in proinflammatory cytokine expression and their release from adipose tissue to the systemic compartment occur not only in response to local obesity-related tissue hypoxia but also in response to systemic hypoxia resulting from reduced pulmonary functions. Nevertheless, it is unclear whether systemic hypoxia exerts additional or multiplicative effects on adipose tissue in patients with COPD and concurrent obesity. Interestingly, similar uncertainties dwell in the search for the potential synergistic negative effects of obesity and obstructive sleep apnea, a condition characterized by repetitive cessation of breathing during sleep and intermittent hypoxia [104]. While the association between sleep apnea and systemic inflammation has been repeatedly reported [105–107], and the effects of intermittent hypoxia on metabolic derangements and systemic inflammation have been documented in animal models [108–111], the topic of adipose tissue response to intermittent hypoxia in patients with sleep apnea is still unexplored. Similarly, the study of the effects of chronic systemic hypoxia related to COPD on adipocyte structure and function represents a major research challenge.

### 5.6. Cross-Talk between Adipocyte Tissue and Lungs: Search for Evidence

Epidemiological evidence that links COPD to obesity and metabolic syndrome, in association with pleiotropic character of most inflammatory mediators, suggests that a physiologically and clinically relevant cross-talk might exist between the lungs and adipocyte tissue. Although such concept has not been yet directly studied in detail, several findings suggest that this hypothesis worth further exploration. First, receptors of two typical adipocyte-derived cytokines, leptin, and adiponectin, are expressed in peripheral tissues including lung [112, 113]. Interestingly, increased leptin expression in bronchial mucosa was observed in patients with COPD, in association with airway inflammation and airflow obstruction [112, 114]. Moreover, leptin receptor polymorphisms were linked to the decline in pulmonary functions, and leptin receptor is considered a novel candidate gene for COPD [115], whereas adiponectin may attenuate allergen-induced airway inflammation and hyperresponsiveness suggesting its potential protective role within the airways [116]. In turn, expression of SP-D, the protein that was originally thought to be restricted to the pulmonary compartment, was recently located in both epithelial cells of multiple mucosal surfaces and in endothelial cells [117]. Interestingly, genetic variations in serum SP-D and BMI are correlated [118], and a negative association between serum SP-D and obesity measures was observed in humans [117]. Nevertheless, the nature of the association remains unknown and additional research is needed in this field.

## 6. Conclusion and Future Perspectives

A new paradigm may emerge in the approach to analyze key question whether adipose tissue contributes to systemic inflammation in such disorders that are associated with both, obesity and systemic hypoxia. Approaching this question is relevant in at least two highly prevalent conditions in human respirology, that is, COPD and sleep apnea. On one hand, in patients with COPD, systemic inflammatory phenotype likely reflects inflammation in the lungs, that is, results from lung-to-plasma spillover of inflammatory mediators. On the other hand, however, obesity-related hypoxia evokes local inflammatory response within adipose tissue *per se*, and systemic hypoxia likely contributes to the adipose tissue inflammation. If so, elevated circulating levels of inflammation-related proteins may reflect also spillover from the adipose tissue to the systemic circulation in patients with COPD and concurrent obesity. Taken together, although current evidence suggests that lung inflammation and spill-over of inflammatory mediators to the systemic compartment represent key mechanisms of systemic inflammation in COPD, both lung and adipose tissue origin of increased levels of circulatory inflammatory proteins likely coincide in some patients. The relative impact of the two mechanisms may depend on at least three factors: severity of pulmonary impairment (i.e., the rate of translocation of inflammatory mediators from the lungs to the systemic circulation), the degree of obesity-related adipose tissue hypoxia, and the severity of systemic hypoxia due to reduced pulmonary functions (Figure 1). Future studies are urgently needed to address these questions.

Ultimately, the crucial task is to develop novel therapies that would be effective in reductions in systemic inflammation, and in reductions of mortality in patients with COPD. In this regard, anti-inflammatory drugs, adipokines, hormones, and appetite stimulants are in the center of research interest. Effective anti-inflammatory therapies carry the potential that they will not only suppress systemic inflammation but also treat comorbid conditions and systemic manifestations of the disease [3]. Future translational and clinical research overlapping respirology and metabolism with the aim to unravel the role of adipose tissue in COPD-associated comorbidities is highly warranted.
